# A patient with a painless neck tumour revealed as a carotid paraganglioma: a case report

**DOI:** 10.1186/1477-7819-12-267

**Published:** 2014-08-20

**Authors:** Barbara Peric, Ziva Pohar Marinsek, Breda Skrbinc, Maja Music, Ivana Zagar, Marko Hocevar

**Affiliations:** Institute of Oncology Ljubljana, Zaloska 2, Ljubljana, 1000 Slovenia

**Keywords:** Paraganglioma, Carotid body tumour, Head and neck surgery

## Abstract

Carotid paragangliomas are usually slowly enlarging and painless lateral neck masses. These mostly benign lesions are recognized due to their typical location, vessel displacement and specific blood supply, features that are usually seen on different imaging modalities. Surgery for carotid paraganglioma can be associated with immediate cerebrovascular complications or delayed neurological impairment.

We are reporting the case of a 36-year-old man who presented with a painless mass on the right side of his neck 11 months after being treated for testicular cancer. After a fine-needle aspiration biopsy, he was diagnosed with a testicular cancer lymph node metastasis. Neck US and fluorine [F-18]-fluorodeoxy-D-glucose (FDG) PET-CT showed no signs of hypervascularity or vessel displacement. The patient underwent a level II to V functional neck dissection. During the procedure, suspicion of a carotid paraganglioma was raised and the tumour was carefully dissected from the walls of the carotid arteries with minimal blood loss and no cranial nerve dysfunction.

The histology report revealed carotid paraganglioma with no metastasis in the rest of the lymph nodes. The patient’s history of testicular germ cell tumour led to a functional neck dissection during which a previously unrecognized carotid paraganglioma was removed.

Surgery for carotid PG can be associated with complications that have major impact on quality of life. A thorough assessment of the patient and neck mass must therefore be performed preoperatively in order to perform the surgical procedure under optimal conditions.

## Background

Paragangliomas (PGs) of the head and neck are highly vascular lesions that can present as a cervical mass mimicking an enlarged lymph node. However, typical localization, vessel displacement and intratumoural flow make the diagnosis of PG highly likely [1].

We are reporting the case of a patient with a history of testicular cancer and a palpable neck mass where initial ultrasound (US) and fine-needle aspiration biopsy (FNAB) provided us with a misleading diagnosis resulting in the improper design of a carotid PG treatment.

## Case presentation

**Figure 1:**
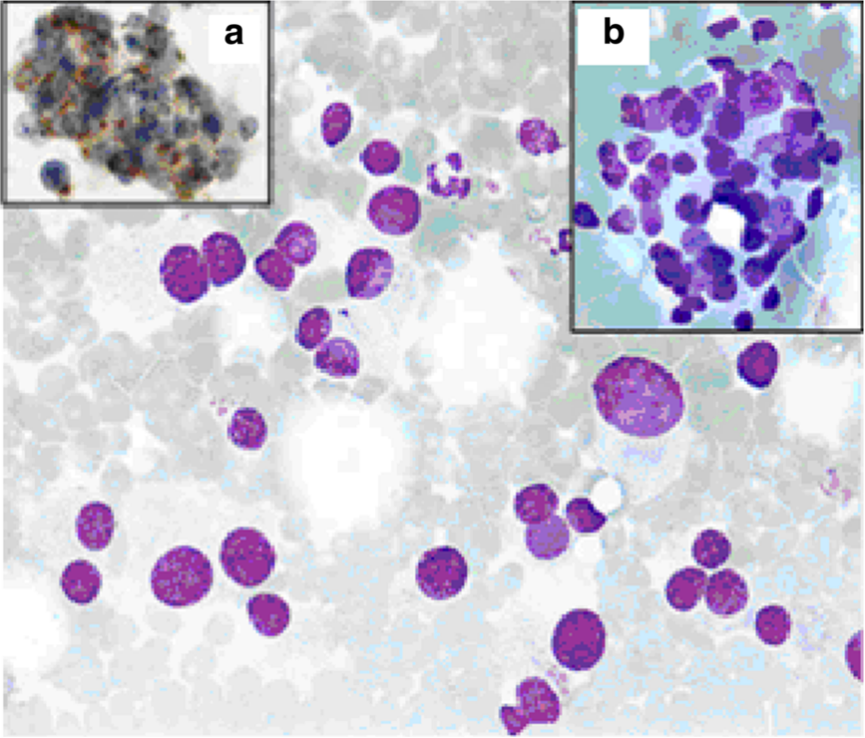
**A FNAB smear of the patient’s tumour.** *FNAB sample featuring dissociated cells with pronounced anisonucleosis. Plasmacytoid shape of some cells is poorly discernable (Giemsa, x60). Panel on the right: a group of tumour cells without a specific organoid structure (Giemsa, x60). Panel on the left: positive immunocytochemical reaction for synaptophysin A, (x60). Reaction was performed after the diagnosis of PG was already known.

**Figure 2:**
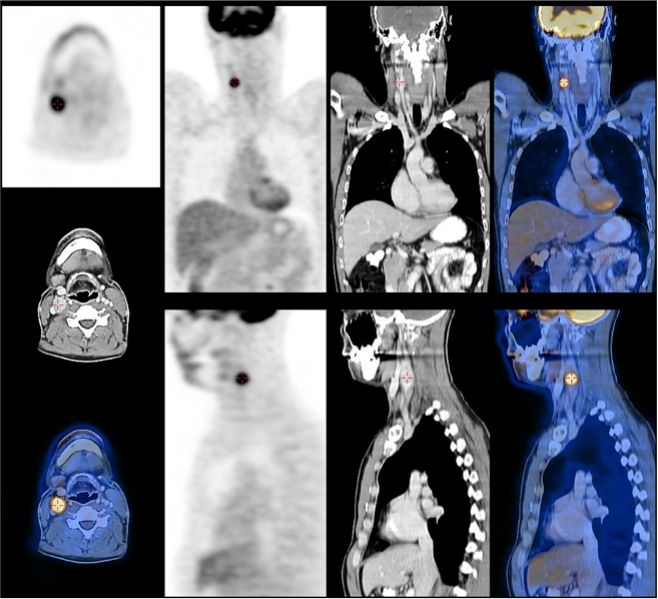
**A positron emission tomography-computed tomography (PET-CT) scan showing the tumour of the neck region III with increased uptake.**

A 36-year-old man presented with a newly discovered mass on the right side of his neck. A year before, he had been treated for testicular cancer classified as a stage I combined germ cell tumour (pT2N0M0). After orchidectomy, the patient was engaged in active follow-up.A few days prior to his regular follow-up visit, he noticed a neck mass. He had no additional complains. Abdominal and testicular US scans, a chest X-ray and blood tests were normal at the time. On clinical examination, a palpable, 2 × 1.5 cm tumour on the right side of his neck was detected. The patient was sent for a FNAB. The smear was described as poorly cellular, composed predominantly of naked nuclei in a haemorrhagic background. The majority of the cells were small, with round nuclei, mixed with some larger cells, which gave the sample an appearance of obvious anisonucleosis. Cytoplasm was seen only in a few cells and gave cells a plasmacytoid appearance. The sample was diagnosed as a lymph node metastasis of a teratomatous component of the germinal tumour or of another primary tumour. Figure 1 represents a FNAB smear of the patient’s tumour.An ear, nose and throat specialist did not discover any suspicious lesions. A neck US scan revealed an enlarged, round lymph node of the neck region III enclosing a carotid artery bifurcation described as metastatic and some additional enlarged submandibular lymph nodes, which were less suspicious. The patient underwent a positron emission tomography-computed tomography (PET-CT) scan, which revealed a 2.1 × 1.7 cm large pathological lymph node with a standard uptake value (SUV) of 10.6 in the neck region III and two slightly enlarged lymph nodes in the left paraaortal space with a maximum SUV of 5.7 (Figure 2).

At the multidisciplinary meeting, the patient’s tumour was recognized as a metastatic lymph node and a level II to V functional neck dissection was proposed.

During the operative procedure, the surgeon noticed that the tumour was situated at the carotid artery bifurcation and was partially enclosing the walls of the internal and the external carotid artery. Suspicion of carotid PG was raised and the tumour was carefully dissected using bipolar cautery from the walls of both arteries with minimal blood loss. All lymph nodes of levels II to V were dissected with preservation of the XI cranial nerve, sternocleidomastoid muscle, the internal jugular vein as well as the supraclavicular sensory nerves. The exposure allowed visual control of the bifurcation, and the external and internal carotid artery during all stages of the carotid PG dissection.

The histology report revealed 2 × 1.5 cm carotid PG without any lymph node metastasis. No signs of cerebral ischaemia or cranial nerve deficits were recorded during the postoperative period. After a short recovery, the patient continued with his regular follow-up visits.

## Discussion

PGs are rare highly vascular tumours that arise from paraganglia. Paraganglia are derived from neural crest cells and can be found in nerve perineurium or vascular adventitia from the skull base to the pelvic floor [1–3]. Head and neck paraganglia are associated with the parasymphetic nervous system but in the chest and abdomen these formations are associated with the sympathetic nervous system and secrete catecholamines [4]. Acting as chemoreceptors and mediating the ‘fight-or-flight’ response, they play a crucial role in providing acute organismic homeostasis [5].

PGs are mostly benign neoplasms as less than 10% have been described to be malignant [2]. These slow-growing tumours cause symptoms by compressing adjacent structures [4]. In adrenal medulla they are referred to as pheochromocytomas [5].

Rare head and neck PGs represent 0.6% of all head and neck tumours [2, 4]. They can occur sporadically or as inherited, familial tumours. Patients with multiple endocrine neoplasia type 2 (MEN 2), von Hippel-Lindau (vHL) disease or neurofibromatosis type 1 (NF1) are prone to head and neck PG. Approximately 30% of apparently sporadic head and neck PGs are associated with germ line mutations in the mitochondrial complex II genes. The described mutations in *SDHB*, *SDHC* and *SDHD* genes cause one out of four paraganglioma syndromes with similar clinical features [6].

Nomenclature of head and neck PGs is sometimes confusing but currently they are referred to by anatomical location: carotid PG, jugulotympanic PG and so on. Their gender distribution varies with male preponderance of carotid and female of vagal PGs [4].

Carotid PGs are usually painless, slowly enlarging lateral neck masses. A physical examination often reveals a rubbery, non-tender mass along the anterior border of the sternocleidomastoid muscle that is more freely movable horizontally than vertically (‘Fontaine’s sing’) because of the adherence to the carotid artery. Sometimes a carotid bruit or a pulsatile character of the tumour can be found. On rare occasions carotid PG can produce a neurologic deficit of the local cranial nerves, with the X cranial nerve being the one most often involved [1]. Besides lymphadenopathies, the differential diagnosis of a lateral neck mass includes brachial cleft cysts, salivary gland tumours, schwanomas and aneurysms of the carotid artery [6, 7].

Metastatic involvement of neck lymph nodes is usually confirmed with FNAB. In the case of the carotid PG, FNAB is unnecessary since the diagnosis can be made on the basis of imaging procedures. It is also believed that the procedure may cause a haemorrhage into the tumour or damage to the carotid vessel wall and thus have potentially life-threatening implications. However, if clinical presentation of the tumour is not straightforward, FNAB may be used but results are usually difficult to interpret [7–9]. The cytological appearance can easily be mistaken for a metastatic tumour due to the high variant morphologic behaviour of neouroendocrine tumours [8]. In the case of our patient, the cells with moderate or no cytoplasm, and with some nest arrangement were described in the cytological specimen, which could indicate chief cells in paraganglia with nests corresponding to Zellballen seen in the case of carotid PG [7].

An additional difficulty was the patient’s history of a compound germ cell tumour, which may contain a teratomatous component with a somatic-type malignancy of a neuroendocrine nature. The presence of atypical epithelioid cells arranged in papillary or glandular structures in a haemorrhagic background on FNAB has been previously reported as misleading in the diagnosis of germ cell tumours [10]. In our case, similar FNAB characteristics led to the diagnosis of metastatic involvement of the lymph node with teratoma or tumour of unknown origin.

The first imaging procedure that enables us to determine the nature of a neck tumour is usually US. In the case of carotid PG, color duplex US may show a highly vascular, solid hypoechoic lesion at the carotid bifurcation [1, 8, 9]. In our case, US showed a 2 cm, round, heterogenic lesion just above the carotid artery bifurcation with hypoechoic parts, which grew around the internal and external carotid artery. Hypervascularity was not observed. The tumour was once again described as a metastatic lymph node.

The patient underwent a fluorine [F-18]-fluorodeoxy-D-glucose (FDG) PET-CT scan. Two suspicious lesions were described: a 2 cm tumour of the neck region III and a 1 cm tumour under the left kidney. Both lesions had increased SUV. On the CT scan obtained during PET-CT, two enlarged lymph nodes in the retroperitoneal space were seen, which only increased the suspicion of metastatic testicular cancer. The appearance of the neck tumour on the CT scan did not raise the suspicion of carotid PG.

Although PET-CT with FDG has proved to be a useful imaging modality for follow-up of patients with seminomas, it is not recommended as a first-line examination in PG imaging since it depicts a multitude of neoplastic and non-neoplastic processes [11].

Most authors recommend only CT or magnetic resonance imaging (MRI) as a method of choice since it enables us to define the location and extension of the tumour [1, 3]. The CT appearance usually depicts the hypervascular nature of the PG, which, in combination with typical location and characteristic splaying of the carotid bifurcation, suggests a specific diagnosis of a carotid PG. MRI provides even more accurate information than CT due to better soft tissue resolution. On MRI, the lesion exhibits low signal intensity on T1- and proton density-weighted images and high signal intensity on T2-weighted images. Multiple areas of high and low signal intensity, the so-called ‘salt-and-pepper appearance’ can be seen within the lesion, representing high and slow flow. The combination of intratumoural flow, typical localization and vessel displacement also rules out most other diagnoses [1].

A diagnosis of neck metastasis led to a functional neck dissection in our patient. Functional neck dissection is a recommended procedure for patients with cervical lymph node metastases of testicular cancer [12]. In the case of carotid PG, it is performed only when lymph node metastases of malignant PG are demonstrated [13].

PGs can be treated by a complete resection, moderate-dose radiotherapy, stereotactic radiosurgery, permanent embolization or even observation [1–3, 9, 12, 14]. Surgeons base their decision to resect a PG with acceptable morbidity on the tumour size, extension, localization and possible multicentricity. Shamblin’s classification is used to describe the difficulty of carotid PG resection. Tumours localized between the internal and external carotid artery that are easily dissected are type I. Tumours described as type II are adherent or partially surrounding the vessels and type III tumours are those that completely encase the carotids [14].

Surgical resection with acceptable operative risk was traditionally recommended in the case of type I and II tumours since all tumours were believed to become symptomatic eventually and some 5 to 7% of carotid PGs were shown to be malignant at the time of presentation [2, 9].

The first step of carotid PG removal consists of the dissection of the common and internal and external carotid arteries as far as the superior and inferior pole of the tumour. From the periphery, the dissection is continued to the carotid bulb with careful isolation of the XII, X, and XI cranial nerves. The advancement from the carotid bulb to the carotid bifurcation constitutes the greatest risk of arterial rupture. When carotid artery replacement is necessary, ligation and section are performed after heparinization, followed by immediate anastomosis with the assistance of a vascular surgeon. The clamping time should not exceed 25 minutes [15]. Since surgery for carotid PG should be performed under conditions allowing vascular surgery, certain authors advise against resection in the case of incidentally discovered PG during neck exploration [13]. However, exposure of carotid vessels during functional neck dissection is adequate for the necessary vascular interventions.

Surgical resection of carotid PG poses a risk of immediate and late complications. According to Makeieff *et al*. the rate of surgical complications depends on how challenging the case is. Permanent nerve palsy and vascular complications are described in 2.3% of type I/II tumours and in 35.7% of type III tumours [13]. Surgical procedure can also be complicated by profuse bleeding due to the high vascularity of the PGs. A recent retrospective study comparing surgical control of carotid PG to radiotherapy described a 3% risk of permanent stroke, 1.4% risk of bleeding and mortality of 1.3% due to postoperative complications [16]. In a review of surgical treatment from a single institution, Amato *et al*. described transient cerebral ischaemia in 6% of cases and 26% of postoperative cranial nerve palsies. Impairment of the X cranial nerve is the main cause of the morbidity after the surgical resection and was described in a study by Amato *et al*. in 9% of cases [17]. Other publications report about less than 5% of cerebrovascular complications and approximately 20% of permanent cranial nerve impairment after the surgical resection [1].

In the case of clearly unresectable carotid PG, multiple PGs or with patients with poor health, radiotherapy should be considered. Long-term control rates of 96% have been reported [15]. After radiotherapy, tumours rarely dissolve completely and local control usually means stability or regression with no progression but also no improvement of neurologic symptoms [9]. Retrospective analysis of both treatment possibilities pointed out avoidance of immediate complications with the use of radiotherapy. Authors have proposed that surgery should be reserved for unilateral tumours with low risk of nerve palsy and cases of malignant or secreting tumours [16].

## Conclusions

Complete resection of the carotid PG, assessed as a Shamblin type II tumour, with no cerebral vascular accidents or nerve impairment was achieved during functional neck dissection for supposed metastatic lymph node involvement. The case shows that previous cancer treatment should be kept in mind when considering all differential diagnoses of a neck tumour, but at the same time our decisions about the usage of diagnostic tools and result interpretation should not be based solely on the patient’s history. Surgery for carotid PG can be associated with complications that have major impact on quality of life. A thorough assessment of the patient and neck mass must therefore be performed preoperatively in order to perform the surgical procedure under optimal conditions.

## Consent

Written informed consent was obtained from the patient for publication of this case report and any accompanying images. A copy of the written consent is available for review by the Editor-in-Chief of this journal.
